# Predicting Impacts of Climate Change on *Fasciola hepatica* Risk

**DOI:** 10.1371/journal.pone.0016126

**Published:** 2011-01-10

**Authors:** Naomi J. Fox, Piran C. L. White, Colin J. McClean, Glenn Marion, Andy Evans, Michael R. Hutchings

**Affiliations:** 1 Animal Health, Scottish Agricultural College, Edinburgh, United Kingdom; 2 Environment Department, University of York, York, United Kingdom; 3 Biomathematics and Statistics Scotland, Edinburgh, United Kingdom; University of Liverpool, United Kingdom

## Abstract

*Fasciola hepatica* (liver fluke) is a physically and economically devastating parasitic trematode whose rise in recent years has been attributed to climate change. Climate has an impact on the free-living stages of the parasite and its intermediate host *Lymnaea truncatula*, with the interactions between rainfall and temperature having the greatest influence on transmission efficacy. There have been a number of short term climate driven forecasts developed to predict the following season's infection risk, with the Ollerenshaw index being the most widely used. Through the synthesis of a modified Ollerenshaw index with the UKCP09 fine scale climate projection data we have developed long term seasonal risk forecasts up to 2070 at a 25 km square resolution. Additionally UKCIP gridded datasets at 5 km square resolution from 1970-2006 were used to highlight the climate-driven increase to date. The maps show unprecedented levels of future fasciolosis risk in parts of the UK, with risk of serious epidemics in Wales by 2050. The seasonal risk maps demonstrate the possible change in the timing of disease outbreaks due to increased risk from overwintering larvae. Despite an overall long term increase in all regions of the UK, spatio-temporal variation in risk levels is expected. Infection risk will reduce in some areas and fluctuate greatly in others with a predicted decrease in summer infection for parts of the UK due to restricted water availability. This forecast is the first approximation of the potential impacts of climate change on fasciolosis risk in the UK. It can be used as a basis for indicating where active disease surveillance should be targeted and where the development of improved mitigation or adaptation measures is likely to bring the greatest benefits.

## Introduction

Fasciolosis is responsible for economic loss in most regions of the world where sheep and cattle are reared [1]. It is a physically and economically devastating disease; heavily infected hosts may die, those with lighter infections may suffer inhibited growth and reduced production efficiency, while the detection of pathological lesions lead to invariable liver condemnation [1]–[3].

Distributions of the free living stages and the intermediate molluscan host, *Lymnaea truncatula*, are dependent on a range of climatic factors. A temperature range of 10–25°C is needed for both *F. hepatica* larvae and *L. truncatula* development with developmental rates temperature dependent within this restrictive range [3]. High levels of moisture are also required, with both *F. hepatica* and *L. truncatula* being vulnerable to desiccation. Ultimately the relationship between rainfall and temperature is the primary determinant of transmission efficiency [4].

As with many parasites there is a distinct seasonal pattern in fasciolosis outbreaks, [5], [6] with two key periods of infection, summer and winter. Summer outbreaks occur when snails are infected in late spring and early summer, with infection maturing during the summer and disease levels peaking during the late autumn/winter period [1], [6]. Winter infection occurs when eggs excreted during unfavourable winters commence development in early spring, once suitable conditions are encountered, [2], [7], with infection typically evident in the host from July to October [8].

Fasciolosis has increased in some EU member states by up to 12 fold in recent years [4], and there is evidence to suggest that it is increasing in the UK [9]. There is also anecdotal evidence of an increase in acute fasciolosis in sheep by mid-summer in recent years in parts of the UK [4], suggesting that climate change is already having a measurable influence on disease dynamics through supporting the development of overwintering larvae [10]. Although the increase in fasciolosis in the UK has been attributed to climate change [10]–[12] the link has not been proven due to the paucity of long term studies and lack of consistent disease incidence data [13].

The strong links between climate and fasciolosis levels have facilitated the creation of short term forecasting models. These short-term forecasts can help to predict fasciolosis incidence and severity at local and regional scales, allowing the development and implementation of improved control strategies [4], [14], [15] with strategic chemical use helping slow the development of resistance.

Short-term prediction models for fasciolosis have been created for many regions across the globe including the USA [16]–[18], Africa [14], [19]–[21], Bolivia [22] and the UK [23]. These models are created using various techniques including process-based mechanistic modelling [24], correlative models based on surveillance data [23] or liver condemnations [1], and a current trend towards GIS models [14]–[18], [20].

Of all the forecast models the Ollerenshaw index [23] was the first widely used system to predict acute outbreaks and manage control strategies in the UK. It was developed using *F. hepatica* prevalence data and climate data from farms and meteorological stations from 1948 to 1957 with a seasonal index derived from measured rainfall, number of rain days and potential evapotranspiration. The National Animal Disease Information Service (NADIS) currently provides farmers with short- term forecasts of fasciolosis risk based on the Ollerenshaw index [25].

The emphasis of previous forecast systems has been on predicting risk for the subsequent year to inform the farming industry and facilitate the implementation of appropriate control measures. So far no models have been applied to long-term climate data to illustrate how intensity and distribution may vary over an extended time scale. Showing where the greatest changes are likely to occur will allow surveillance to be most efficiently targeted. The release of UKCP09, with fine scale predictions for a number of climate variables [26], provides the opportunity to make meaningful long term projections. Here we combine a modified Ollerenshaw risk index with the UKCP09 climate data to predict how climate change will influence fasciolosis risk, up to 2070. We also create risk maps for 1961–2005 to illustrate how climate has already influenced risk. We generate risk maps for summer and winter infection risk independently to allow changes in timing of disease outbreaks to be considered. The resultant maps allow comparisons between future risk and risk levels experienced to date, in addition to highlighting the regional differences and expected changes in seasonal patterns of outbreaks.

## Methods

To calculate *F. hepatica* infection risk, the Ollerenshaw index was used, with some slight modifications due to the availability of climate data. This model is dependent on the interactions between rainfall and temperature, with the monthly fasciolosis risk value (Mt) calculated as below [23]:
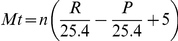



Mt  =  Fasciolosis risk value,

n  =  Number of rain days per month,

R  =  Rainfall (mm/month)

P  =  Potential evapotranspiration (mm/month).

For the calculation of potential evapotranspiration (P), the Hargreaves equation for evapotranspiration was used, where Ra is extraterrestrial radiation [MJ m^−2^ day^−1^] [27], [28]:
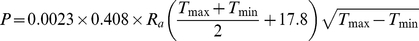



Below we briefly describe the key elements of the Ollerenshaw index. For a full description of the Ollerenshaw index see Ollerenshaw & Rowlands, [23]. An Mt value was first calculated for each month and these monthly values were subsequently summated to give seasonal risk values (summer and winter). Mt was set to zero if the mean monthly temperature was below 10°C, to reflect the development thresholds for both the free living stages of *F. hepatica* and *L. truncatula*. The mean temperature was used as fasciolosis transmission can occur when night time temperatures are below the minimum development threshold of 10°C if they are compensated for by high day time temperatures [12]. When the model was originally created mean temperatures were only above 10°C between May and October, hence only these months were included in the model. As temperatures have increased over the past five decades, and are predicted to continue rising [26], the modified method used here includes all months with a mean temperature greater than 10°C. As with the original model a restriction was applied to the calculation to allow for exceedingly wet years. When the monthly Mt value reaches 100 it can be assumed that the moisture levels, regardless of previous state, have become sufficiently wet to permit parasite development [23]. Consequently, monthly Mt values are capped at 100. The cap of 100 was always reached when number of rain days per month exceeded 18.

For the risk maps, the seasonally summated summer and winter Mt values were grouped into four risk categories following Ollerenshaw & Rowlands [23]: Mt <300, little or no disease; 300< Mt  = 400, occasional losses; 400< Mt  = 474, disease prevalent; and Mt >474, serious epidemic.

### Climate Data

#### Past

For the calculation of past fasciolosis risk, climate data were obtained from the UKCIP gridded datasets [26]. These data are based on interpolations of surface observations, which provide a grid of values for 5 km squares across the UK. Each value represents a point at the centre of the 5 km square and the Ordnance Survey National Grid was used to identify the grid cells. Monthly data from 1970 to 2006 were used, with the following climate variables: minimum, maximum and mean monthly temperature (°C), number of rain days per month (>1 mm), and monthly rainfall (mm).

#### Future

For predicting future fasciolosis risk, the UKCP09 climate data were used from the HadCM3 climate model [26], using monthly climate change averages at 25 km square resolution for six 30 year time periods: 2010–2039, 2020–2049, 2030–2059, 2040–2069, 2050–2079, 2060–2089. In the analysis these time periods are referred to as 2020, 2030, 2040, 2050, 2060 and 2070 respectively. The medium emissions scenario (IPCC SRES:A1B) was used. The climate variables used were: mean temperature (°C), mean daily maximum temperature (°C), mean daily minimum temperature (°C), precipitation (mm/day), cloud cover (%) and relative humidity (%).

#### Comparing past and future risk

For comparison between past and future risk, long term climate averages were used. Past Mt risk was calculated using long term average data from 1961–1990. These data were based on the HadRM3 regional climate model and were downloaded at a resolution of 25 km squares [26]. The future long term average was calculated using the modified Ollerenshaw method and average climate data from 2030–2070, at a resolution of 25 km squares.

### Modification for Future Projection

As there are no projections for rain days, a model was created to calculate a surrogate rain days value using available climate parameters. The model was created using the 1961–1990 long term monthly averages (LTAs), from the UKCIP 25 km gridded data set covering the whole of the UK and based on the HadRM3 regional climate model [26]. This dataset included the variables: cloud cover (%), maximum temperature (°C), minimum temperature (°C), mean temperature (°C), relative humidity (%), rainfall (mm/day) and number of rain days (days/month). Data from all parts of the UK for all months of the year were included.

To determine a surrogate value for rain days using other climate variables a generalized additive model (GAM) was used as this allows the modelling of non linear relationships [29] with the smoothing functions enabling non-parametric response curves to be fitted separately to each predictor variable [30]. The GAM function from the mgcv library in R was used [31].

The GAM was built using the 1961–1990 LTA climate data [26]. The climate data were split spatially with the training data set comprising a randomly selected 80% of the points (n = 4215). The variables with collinearity (minimum temperature and maximum temperature) were removed and the remaining variables were pre-screened by fitting GAM models to each variable in isolation. The remaining parameters included in the model were selected using generalised cross validation (GCV) [31]. Parameters included were number of rain days, mean temperature, cloud cover, rainfall and humidity with the final model explaining 90.2% of the deviance (P<0.0001 (for all variables), GCV  = 1.117).

**Figure 1:**
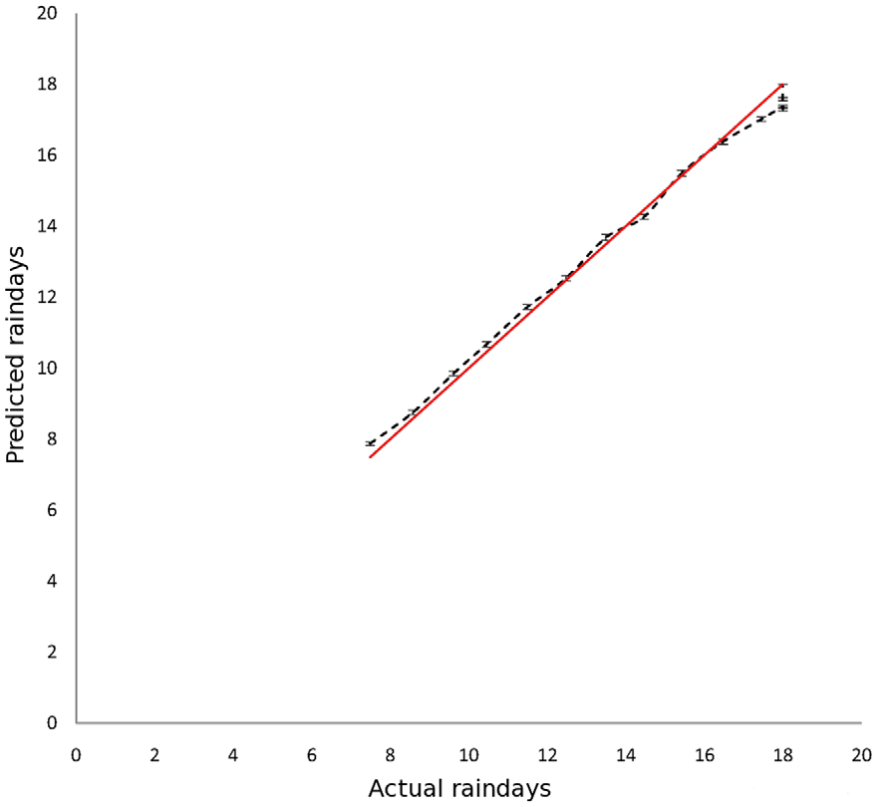
The number of rain days predicted using the GAM against the actual number of rain days (dashed line) (± coefficient of variation). The line of perfect correlation is also shown (solid line).

The predictive power of the model was first evaluated using the test dataset, comprised of the remaining 20% of the 1961–1990 long term average climate data (n = 1054). Fig. 1 shows the predicted rain days and the actual rain days put into bins of integer rain days, ± the coefficient of variation. The Ollerenshaw model is capped when rain days exceed 18, as above this value the area is considered moist enough to allow for maximal larval development, and any higher value would not increase infection risk any further. This validation suggests that the model is accurate when extrapolating spatially, indicating the models reliability when applied to novel climatic conditions (Fig. 1). To substantiate the accuracy when extrapolating temporally the model predictions were tested on the 1991–2000 monthly gridded data-sets [26], with 10 year monthly averages for all parameters. Despite applying the model to data of a different temporal and spatial resolution to the training data-set, it still provides a reliable surrogate for rain days, with the model explaining 92% of the variation (R2 = 0.92, df  = 1210, mean predicted – mean actual  = 0.6 rain days).

In a further validation of the model, using the 1961–1990 test dataset (n = 1054), the Mt values were first calculated using the original Ollerenshaw index, and then calculated using the modified Ollerenshaw index, with the GAM providing a surrogate value for rain days. There was no significant difference in Mt calculated using the original and modified approach (Paired t-test, t = 1.42, df  = 1052, p-value >0.1) for the historic period.

The modified Ollerenshaw index was therefore used to calculate future fasciolosis risk.

### Spatial Analysis

**Figure 2 pone-0016126-g002:**
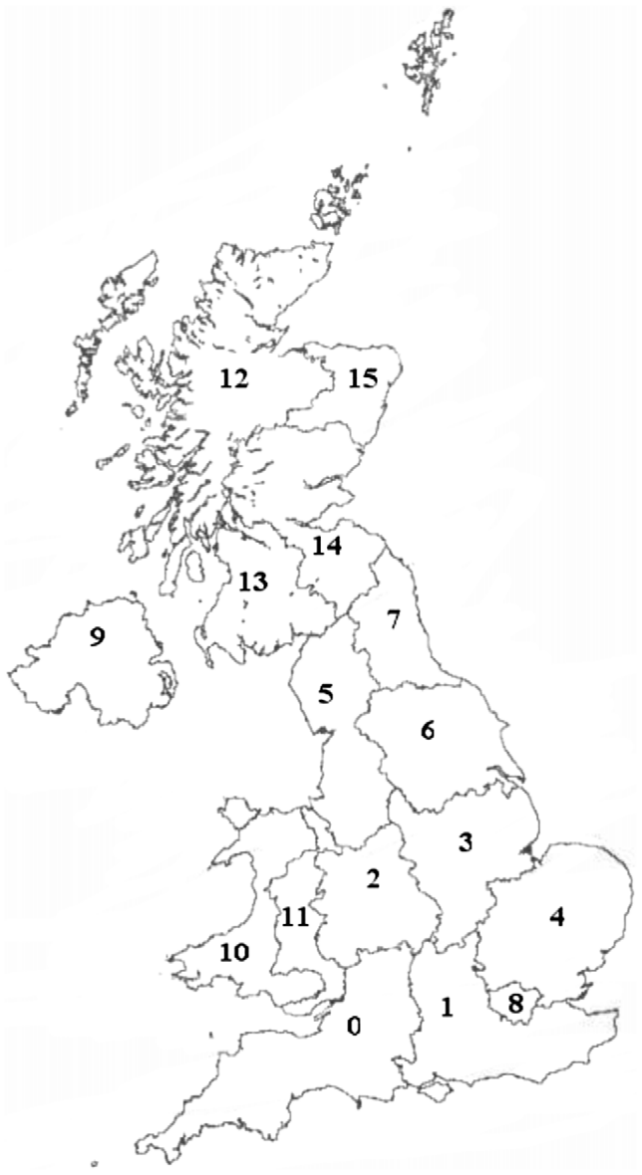
The regions of the UK used in comparing past and future risk. Region details are shown in Table 1.

Fine scale risk maps for past and future Mt were generated using ArcGIS. However, comparisons between past and future risk were made at the scale of the regions shown in Fig. 2 and Table 1. These follow administrative boundaries with GIS layers downloaded and merged from EDINA [32].

**Table 1:** The 15 regions of the UK used in comparing past and future risk.

Code	Region
0	South West
1	South East
2	West Midlands
3	East Midlands
4	East of England
5	North West
6	Yorkshire and The Humber
7	North East
8	London
9	Northern Ireland
10	West Wales and The Valleys
11	East Wales
12	Highlands and Islands
13	South Western Scotland
14	Eastern Scotland
15	North Eastern Scotland

England  = 0–8, Northern Ireland  = 9, Wales  = 10–11, Scotland  = 12–15.

## Results

### Reconstructing Past Risk

**Figure 3:**
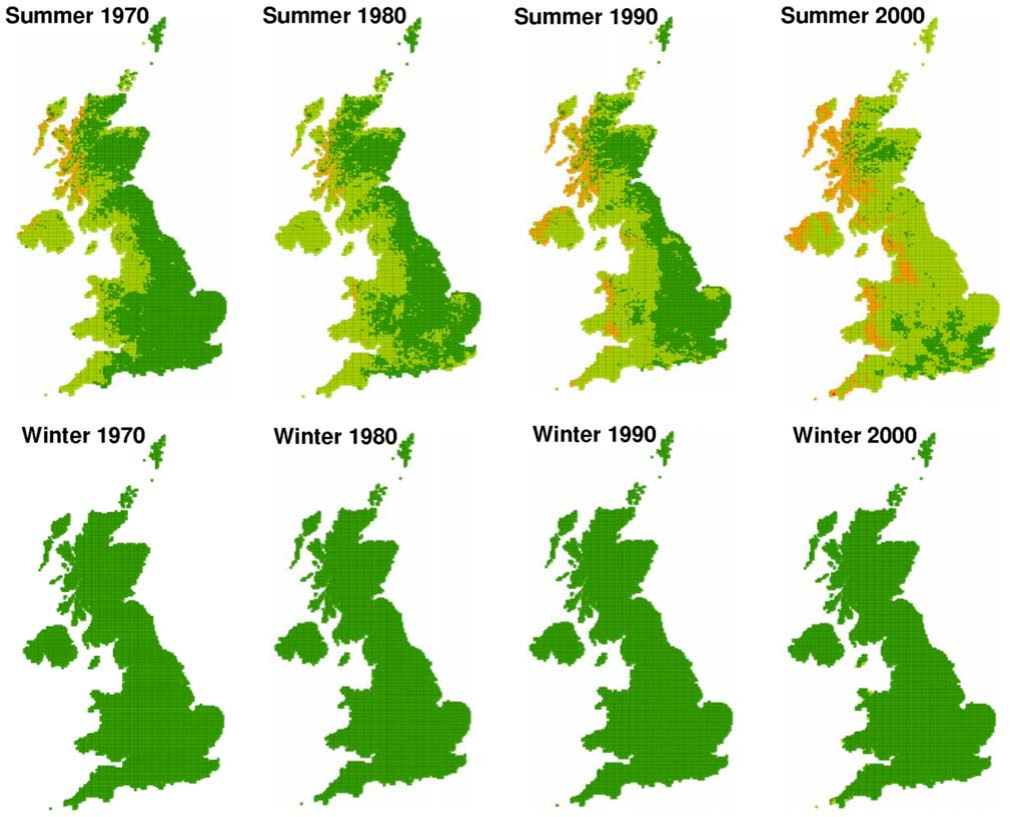
Past change in fasciolosis risk. Decade averages of summer and winter *F.hepatica* risk across the UK at a resolution of 5 km squares, 1970–2006. Risk categories are based on those used by Ollerenshaw & Rowlands [22]: Little or no disease: Mt <300 (dark green), occasional losses: 300< Mt  = 400 (light green), disease prevalent: 400< Mt  = 474 (orange), serious epidemic: Mt >474 (red).

The average fasciolosis risk from summer infection has increased across the past four decades. The maps indicate that in the 1970s a majority of the UK was fasciolosis free, however by 2000 most of the UK was suffering occasional losses, with disease being prevalent in large sections of West Wales and Scotland (Fig. 3). This pattern of increase across the UK echoes the trends reported from passive surveillance records [9], [11], [13], [33]. There has been little change in risk from overwintering larvae, however by 2000, there are areas of Cornwall and the Welsh west coast showing occasional losses, as minimum development threshold temperatures have been exceeded in recent years.

**Figure 4:**
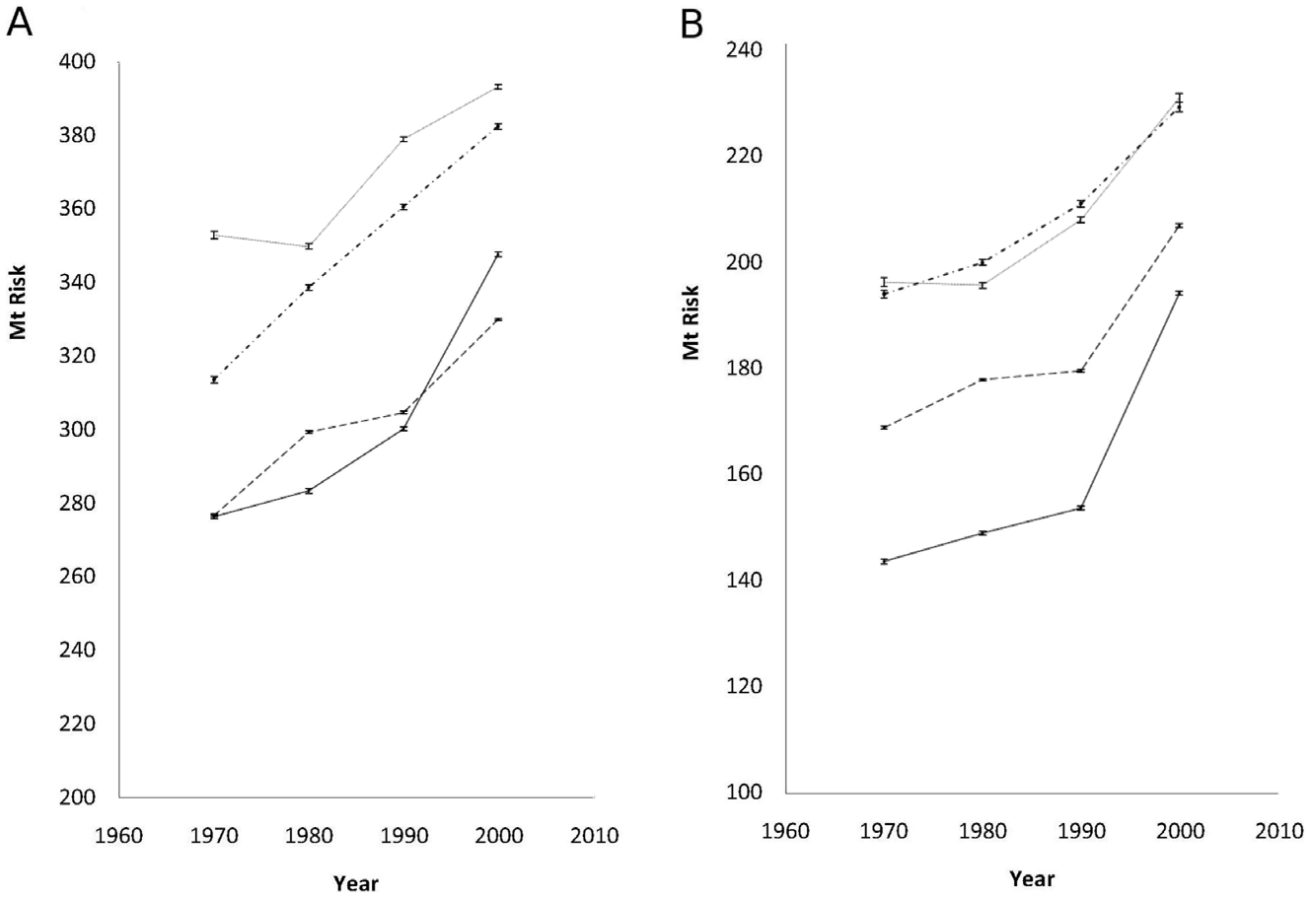
Past trends in fasciolosis risk. Change in *F. hepatica* risk for England (dashed line), Scotland (solid line), Wales (dashed and dotted line) and Northern Ireland (dotted line), 1970–2006 (± SE) for a) summer and b) winter.

There has been a steady rise in *F. hepatica* risk in all parts of the UK (Fig. 4). Northern Ireland and Wales have so far experienced the highest levels of fasciolosis risk, with Scotland showing higher summer levels than England since the 1990s (Fig. 4a). The mean risk from overwintering larvae has increased in all parts of the UK (Fig. 4b). This matches reported changes in timing of fasciolosis infection, with outbreaks occurring earlier in the year due to the ingestion of overwintering larvae [4]. Despite the steady increase in winter risk index, a majority of the UK remains in the lowest risk category.

### Predicting Future Risk

**Figure 5:**
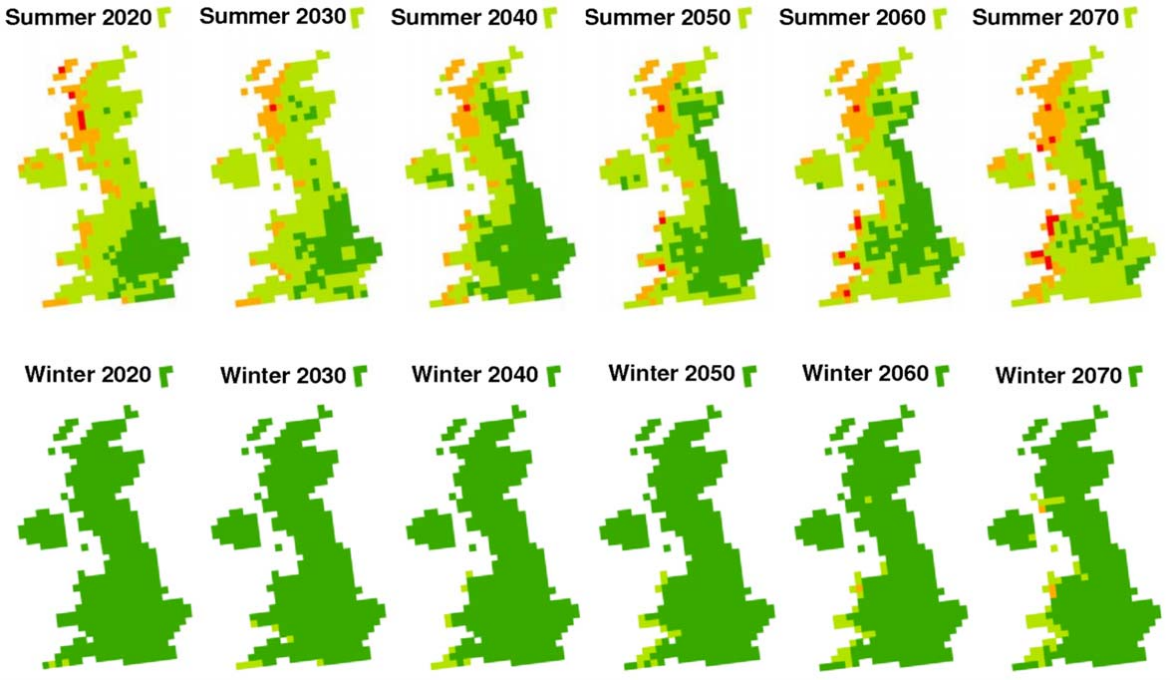
Projected change in fasciolosis risk. Summer and winter *F. hepatica* risk across the UK at a resolution of 25 km squares, 2020–2070. Risk categories are based on those used by Ollerenshaw & Rowlands [22]: Little or no disease: Mt <300 (dark green), occasional losses: 300< Mt  = 400 (light green), disease prevalent: 400< Mt  = 474 (orange), serious epidemic: Mt >474 (red).

The future risk maps show the projected change in fasciolosis risk from both winter and summer outbreaks (Fig. 5). The summer maps illustrate that predicted risk increases in certain areas, with serious epidemics predicted in Wales by 2050. However due to the complexity of future climate change and the long term variation in the system, risk will reduce in some areas and fluctuate greatly over time in others. A steady increase in risk from overwintering larvae is depicted along the West Coast as mean monthly temperatures exceeding 10°C become commonplace, with Wales again being most at risk.

**Figure 6:**
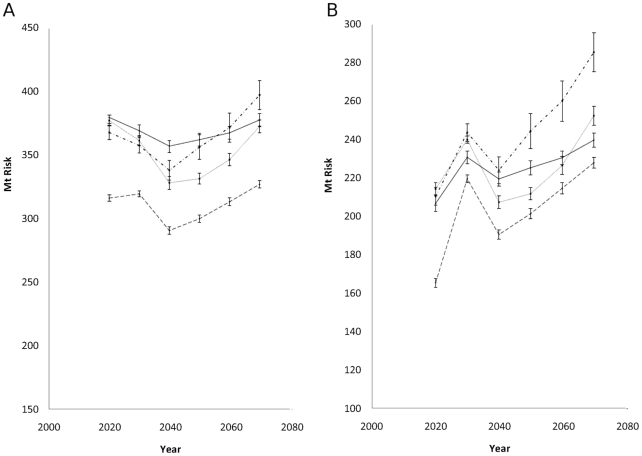
Future trends in fasciolosis risk. Predicted change in *F. hepatica* risk for England (dashed line), Scotland (solid line), Wales (dashed and dotted line) and Northern Ireland (dotted line), 2020–2070 (± SE) for a) summer and b) winter.

In terms of the overall trend in summer risk by country, Wales shows the greatest rise (Fig. 6a). The non linear increase in all regions due to the fall in 2040 echoes a decline in predicted summer rainfall during this time period. There is a projected rise in risk from overwintering larvae across the UK, with the greatest rise being in Wales (Fig. 6b). The steep rise in 2030 is due to high levels of predicted rainfall.

### Comparing Past and Future Risk

**Figure 7:**
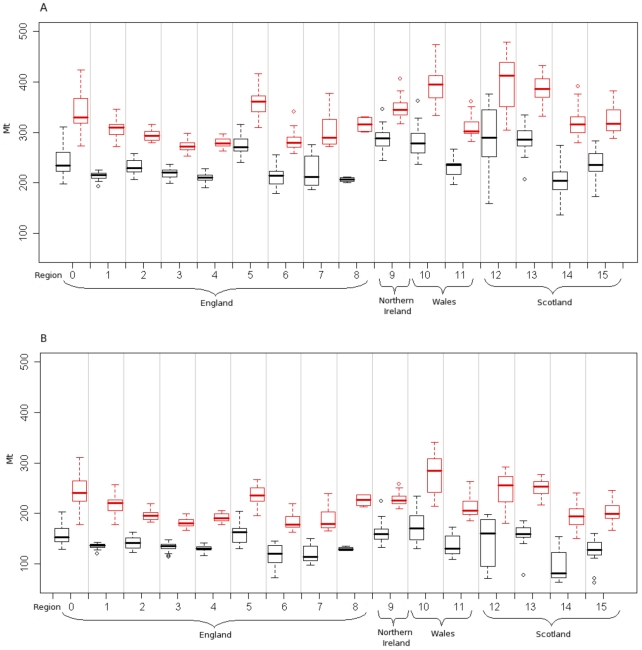
Comparing past and future risk. *F. hepatica* risk for each region for both 1961–1990 (black) and 2030–2070 (red) long term averages, for a) summer and b) winter. For region codes see Fig. 2 [Fig pone-0016126-g002].

The mean fasciolosis risk in each season is predicted to be higher in the future than the past for all regions of the UK (Fig. 7), with the highest overall predicted risk being in West Wales (region 10). There is intra-region variability predicted, with the Highlands of Scotland (region 12) showing the greatest range in risk.

## Discussion

Over the past four decades fasciolosis risk has spread across the UK from a restricted distribution in 1970, with only occasional cases seen in the west, to the current levels where large swathes of the UK are seeing regular outbreaks. The increase that is evident in the 1970–2006 risk maps is consistent with empirical data [9], [11], [34] and provides further evidence that the changes in fasciolosis in the UK are indeed climate-driven. The reliability of the Ollerenshaw index is further substantiated by the agreement between the map of current risk levels and the latest fasciolosis prevalence data [33], however the lack of long term, fine scale prevalence data makes quantitative validation of the model impossible.

Although liver fluke has been having a big impact on farms in recent years, according to the Ollerenshaw index we are yet to experience extended large scale epidemics. It is predicted here that the UK may experience unprecedented levels over the next 60 years, with the overall future risk from fasciolosis set to be higher than the past risk for all regions of the UK. The future maps show that serious epidemics are expected to be the norm by 2020 in parts of Scotland, and by 2050 in parts of Wales. Due to the coarser scale of the future risk maps, localised areas of serious epidemic could be more easily obscured; for a serious epidemic risk to show on the map a high increase in the average risk index is required throughout a 25 km square over a 30 year period.

The model drivers are rainfall, temperature, and the interactions between them. Temperature has the major impact in areas where the mean temperature is raised above the 10°C threshold and rain is not restrictive. This is especially pertinent in the Scottish Highlands in the summer months. The rise in temperature also accounts for the change in risk from overwintering larvae with the increased temperature in the winter months permitting their survival and development, resulting in a spread of risk heading north along the West Coast. Where temperatures are already above the development threshold of 10°C the primary driver becomes changing rainfall patterns. Risk is highest in areas with extended high annual rainfall, high soil moisture and surplus water. Where high temperatures are combined with decreased rainfall the resulting soil moisture deficits will threaten the intermediate hydrophilic stages of the parasites life cycle. This accounts for the trend seen along the East Coast of the UK where future risk decreases from a current level of ‘occasional losses’ to a state of little or no disease by 2040.

Interactions between rainfall and temperature account for the fine scale spatial variation within each region with some locations set to experience diminishing infection levels. For example there is a predicted decrease in the warmer and drier parts of the South West, although this decrease is not enough to drive an overall decline at the regional level. The highest within region variation is seen in the Scottish Highlands, where the levels of spatial climatic variability are highest. The lowest variation is in the East Midlands and East of England, where rainfall and temperature levels are more consistent across each region. Although intra-region variation is of interest, caution must be exercised when applying the Ollerenshaw index at too fine a spatial resolution. At the local level a myriad of non climatic factors begin to exert the dominating influence over *F. hepatica* survival and development [14], [17].

In addition to spatial variation, there will be temporal variation in infection risk across the UK. Overall summer infection risk is expected to decrease below current levels in 2040. This is a consequence of the projected decrease in summer rainfall levels at this time, antagonised by ever increasing summer temperatures, resulting in decreased moisture availability.

To date there has been little increase in risk from overwintering larvae, as the minimum development threshold is still not consistently reached across a majority of the UK. However the maps show a few pockets in the South West and along the Welsh coast where risk from occasional losses has begun to appear. This phenomenon is already being realised on the ground with reports of changing timing of infection [4]. Winters are predicted to become even milder and more months will have a mean temperature of above the 10°C development threshold. As a consequence, outbreaks in late spring/early autumn can be expected. These will be restricted initially to the far south, but will spread slowly north over time. With some parts of the UK experiencing longer development windows, fasciolosis infection could extend from being a seasonal to a year-round threat. Active disease surveillance should be focused in the South and West to capture changes in timing of infection, as this will impact on control strategies such as timing of anthelmintic administration. Surveillance should also be targeted in the areas of Scotland and Wales where serious epidemics are predicted.

Due to the limited scope of current climate projection scenarios, it is not possible to determine the impacts of short term weather fluctuations on infection levels. Increases in extreme events have been forecast [26] and extreme fluctuations could be inimical to fluke survival. If the predicted combination of high temperatures and droughts are realised, the larvae and intermediate hosts could succumb to desiccation. Conversely, drought years can result in increased fasciolosis risk. Following the dry summers of 1959 [8] and 2003 [11], there were exceptionally high levels of fasciolosis in livestock. The losses following dry periods are a consequence of stock being forced to graze verdant flukey areas as water levels recede and metacercariae-free herbage becomes sparse [8]. In contrast, extensive rainfall can be detrimental to transmission as snails and fluke larvae can be washed away by large quantities of rain [3].

A changing climate is also likely to have wider impacts, affecting farming behaviour [35], [36] and land suitability for different farming practices. There will also be direct impacts of climate on the hosts of infection [37], [38] combined with increased anthelmintic resistance [39] exacerbating infection risk. Due to these other factors, together with the gap between the spatial and temporal scales of climate modelling and the real scales at which transmission dynamics exist, the forecasts presented here can only be considered indicative. Nevertheless, our climate-driven risk maps closely match past changes in the infection, suggesting that climate is the dominant driver of these changes.

Regular reviews of the fasciolosis risk predictions should be carried out as long-term climate projections become ever more refined. However, modifications to the broad risk patterns are likely to be relatively minor. It is clear that *F. hepatica* infection in Britain is likely to expand both geographically and in terms of severity, with regions such as West Wales set to experience unprecedented levels of disease outbreaks. Due to the complexities of climate change and its impacts on *F. hepatica* transmission there will be spatio-temporal variation in parasite risk, with some areas experiencing diminishing disease levels. Our projections show where limited resources should be focussed and surveillance should be targeted. Ultimately, data from such surveillance will improve our understanding of the impacts of climate change on parasite levels, and provide information which can help us to develop effective mitigation or adaptation strategies.
